# Development and evaluation of an anti-rabies virus phosphoprotein-specific monoclonal antibody for detection of rabies neutralizing antibodies using RFFIT

**DOI:** 10.1371/journal.pntd.0006084

**Published:** 2017-12-21

**Authors:** Jihye Um, Byung Chul Chun, Yeong Seon Lee, Kyu Jam Hwang, Dong-Kun Yang, Jun-Sun Park, Su Yeon Kim

**Affiliations:** 1 Division of Zoonoses, Center for Immunology & Pathology, Korea National Institute of Health, Cheongju-si, Chungcheongbuk-do, Republic of Korea; 2 Department of Epidemiology and Health Informatics, Graduate School of Public Health, Korea University, Seoul, Republic of Korea; 3 Pathogen Resource TF, Center for Infectious Disease, Korea National Institute of Health, Korea Centers for Disease Control and Prevention, Chungcheongbuk-do, Republic of Korea; 4 Viral Disease Research Division, Animal and Plant Quarantine Agency, MAFRA, Gimcheon, Republic of Korea; 5 Infectious Diseases Research Center, Research Institute, National Medical Center, Seoul, Republic of Korea; Kansas State University, UNITED STATES

## Abstract

**Background:**

Rabies is a major public health problem with a fatality rate close to 100%; however, complete prevention can be achieved through pre- or post-exposure prophylaxis. The rapid fluorescent focus inhibition test (RFFIT) is one of the recommended testing methods to determine the production of neutralizing antibodies after vaccination. Here, we report the development of a new monoclonal antibody (mAb) designed to react specifically with *Rabies virus* (RABV) phosphoprotein (P protein), and the evaluation of its applicability to the RFFIT and its effectiveness as a diagnostic reagent for human rabies.

**Methodology/principal findings:**

The mAb KGH P 16B8 was produced to target the P protein of the Korean KGH RABV strain. An indirect immunofluorescence assay (IFA) was conducted to detect various strains of RABV in various cell lines. Alexa-conjugated KGH P 16B8 (16B8-Alexa) was developed for the RFFIT. The IFA test could detect RABV up to a 1:2,500 dilution, with a detection limit comparable to that of a commercial diagnostic reagent. The sensitivity, specificity, positive predictive value, and negative predictive value of the RFFIT using 16B8-Alexa in 414 clinical specimens were 98.67%, 99.47%, 99.55%, and 98.42%, respectively. The results of the RFFIT with 16B8-Alexa were strongly correlated with those obtained using an existing commercial diagnostic reagent (r = 0.995, *p*<0.001).

**Conclusions/significance:**

The mAb developed in this study shows high sensitivity and specificity, confirming its clinical utility with the RFFIT to measure the rabies neutralizing antibody titer and establish a diagnosis in human. Thus, 16B8-Alexa is expected to serve as an alternative diagnostic reagent that is widely accessible, with potentially broad applications beyond those of the RFFIT in Korea. Further studies with 16B8-Alexa should provide insight into the immunological mechanism of the P protein of Korean RABV.

## Author summary

Rabies represents a significant cause of fatality upon presentation of symptoms; however, pre- or timely post-exposure prophylaxis can provide complete protection to a population. The current reagents available for laboratory tests to determine the level of *Rabies virus* (RABV) neutralizing antibodies are not readily accessible in several regions, including Korea, and are associated with time and economic constraints owing to the import process. To resolve these issues, we developed a new monoclonal antibody with a fluorescent marker (16B8-Alexa) that targets the RABV phosphoprotein from a recent Korean isolate. We show its good detection ability, specificity, and sensitivity, demonstrating highly concordant results with those obtained with a standard commercial reagent using over 400 clinical samples applied to a World Health Organization-recommended diagnostic test. Thus, 16B8-Alexa shows great potential to resolve the current limitations in rabies diagnosis and monitoring in Korea, and is expected to serve as a valuable research tool for immunological studies on RABV.

## Introduction

Rabies is the oldest and most fatal viral zoonosis that has been known to humans for at least 2,300 years. This disease remains a significant public health burden, and it is estimated that more than 60,000 people in over 150 countries worldwide die from rabies every year. In particular, Asia and Africa account for more than 95% of the global prevalence of human rabies [1].

Rabies causes inflammation in the central nervous system of warm-blooded animals, including humans, and is usually transmitted to humans through bites or scratch wounds from animals infected with the virus. Approximately 99% of all cases of human rabies are caused by virus transmission from dogs [2], although wild animals such as bats, raccoons, cats, and foxes may also transmit rabies to humans.

Korean cases of rabies in animals were officially documented for the first time by Japanese researchers in 1907 during the Japanese occupation. Rabies cases were variously observed both in humans and animals until 1984, and no case occurred until 1992 as a result of preventive measures taken. However, rabies cases reappeared in 1993 in animals, followed by continuous case reports since then. There was no incidence of rabies in humans from 1995 to 1998, but it reappeared in 1999, with six cases reported from then until 2004 [3]. Among recently isolated strains, the complete genome sequence of the *Rabies virus* (RABV) KGH strain has been determined. KGH was the first human RABV strain isolated in Korea from hair follicles of a rabies patient whose symptoms developed following a raccoon bite in 2001. The entire KGH genome is 11,928 nucleotides in length. In comparison to the other 40 RABV strains whose complete genomes have been sequenced, there is one unique amino acid replacement in the KGH strain in a region of the phosphoprotein (P protein), which is related to STAT1 control. Phylogenetic analysis showed that KGH is most closely related to the NNV-RAB-H strain isolated in India and the transplant RABV serotype 1 strain [4].

Clinical manifestations of rabies have two forms: the furious form and the paralytic form. Approximately 80% of patients with rabies exhibit the furious form (also termed “encephalitic rabies”), which is characterized by prominent autonomic nervous system disorders, such as hypersalivation and sweating. As a classical sign, confusion, aggressiveness, hydrophobia or aerophagia may occur due to infection of the nervous system or hypersensitivity of the sensory organs; moreover, impairment of consciousness, paralysis and multisystem organ failure could result in death [5, 6]. Approximately 20% of patients with rabies show the paralytic form, which present symptoms such as numbness and muscle weakness without accompanying brain inflammation or hydrophobia [5, 6]. This variation makes it difficult to establish a differential diagnosis of rabies from other central nervous system diseases, which can result in a lack or delay of effective treatment, leading to death of the patient [7].

The lethality of rabies is nearly 100% upon presentation of symptoms; however, infection is completely preventable through pre-exposure prophylaxis (PrEP) or timely post-exposure prophylaxis (PEP) [8]. The World Health Organization (WHO) recommends PrEP for people with a high risk of exposure to rabies based on geography or occupation, including laboratory workers, as well as travelers planning to visit rabies high-risk areas and children living in or visiting rabies-affected areas [1]. PEP includes disinfection and treatment of the wound area as early as possible, and administration of a vaccine alone or with rabies immunoglobulin, depending on the type of exposure. The rapid fluorescent focus inhibition test (RFFIT) is considered the gold standard method for determining the level of RABV neutralizing antibodies. The WHO recommends 0.5 international units (IU)/mL by RFFIT as a neutralizing antibodies indicator of adequate vaccination in human at risk for rabies exposure [1].

A rabies antigen-specific antibody is required to detect infected cells through the RFFIT. While antibody reagents for this test are commercially available, these often pose an economic burden and are difficult to distribute widely to the areas that are the most in need. Since the amendments to the Enforcement Regulations of the Medical Device Act came into effect in 2011, it has been increasingly difficult to import RABV antibodies in Korea. Therefore, there is an urgent need for a supply of an alternative antibody that is applicable in diagnostic testing for the detection of RABV antigen and measurement of the neutralizing antibody titer. In addition, although only RABV belonging to genotype 1 has been reported in Korea to date, bat species that are potential hosts of bat-related lyssavirus inhabit Korea, thereby posing an additional risk. Since P protein contains a broadly cross-reactive epitope for all lyssaviruses, monoclonal antibodies (mAbs) targeting P protein have been proven to be potentially useful for diagnosis and serotyping [9]. To this end, in this study, we developed an Alexa-conjugated monoclonal antibody (mAb) that specifically combines with P protein, using the first domestic KGH strain isolated from a human. The utility of this fluorescently labeled KGH P protein-specific mAb was evaluated through its application to the RFFIT, using 414 clinical specimens.

## Materials and methods

### Ethics statement

This study was approved by the Institutional Review Board of the Korea Centers for Disease Control and Prevention (KCDC; IRB No. 2015-07-05-R-A) and the data were analyzed anonymously.

### Target protein expression and mAb production

Total RNA of the KGH isolate was amplified from virus-infected mouse neuroblastoma (N2a) cells (American Type Culture Collection [ATCC] CCL131), using the RNeasy Plus Mini Kit (Qiagen, Hilden, Germany). cDNA was synthesized from viral RNA by reverse transcription-polymerase chain reaction (PCR) and used as template to amplify the KGH P protein-coding sequence. The oligonucleotide was amplified with two primers (P Ab_F: 5ʹ-CAC CAT GAG CAA AAT CTT TGT CAA-3ʹ, P Ab_R: 5ʹ-GCT GGA TAC ATA GCG ATT CAG ATC-3ʹ). The thermocycling parameters were 30 cycles of 30 s at 94°C, 30 s at 60°C, 1.5 min at 72°C, and a final extension for 5 min at 72°C. The 1353-bp PCR products were visualized by agarose gel electrophoresis and purified using the QIAquick Gel Extraction kit (Qiagen, Hilden, Germany). The fragment was cloned into the pBAD202 Topo vector (Invitrogen, Waltham, MA, USA) and expressed in *Escherichia coli*. The pBAD202/KGHP clone insert was verified through sequencing.

Protein expression and mAb production were conducted by Youngin Frontier Co. Ltd. To determine the optimum conditions for the most effective KGH P protein expression, competent-cell type, isopropyl β-d-1-thiogalactopyranoside (IPTG) concentration, temperature, and time were evaluated. The pBAD202/KGHP was transformed into BL21(DE3), BL21(DE3)pLysS, BL21 codon-plus RIL, and Rosetta(DE3) competent cells, respectively (New England Biolabs, Ipswich, MA, USA). IPTG was used to induce protein expression, at concentrations of 0.1 mM, 0.4 mM, and 1 mM. The amounts of proteins expressed under incubation at 37°C for 4h at 200rpm and at 20°C overnight at 110rpm were verified by sodium dodecyl sulfate-polyacrylamide gel electrophoresis (SDS-PAGE). Two buffers, 20 mM Tris and 50 mM NaH_2_PO_4_, were tested to select the optimum buffer condition for purifying the expressed protein by Ni-NTA His-tag affinity chromatography. Protein expression was also verified through SDS-PAGE in this case.

The verified protein was used to produce the specific mAb for KGH P protein (KGH P 16B8) according to the standard protocol for mAb production outlined by Youngin Frontier. To allow for its utilization in the RFFIT, Alexa Fluor 488 fluorescent particles were conjugated with KGH P 16B8 (16B8-Alexa) using the Alexa Fluor 488 protein labeling kit (Molecular Probes Inc., Eugene, OR, USA) according to the manufacturer’s instructions.

### Reactivity test and limit of detection

An indirect immunofluorescence assay (IFA) was conducted to test whether KGH P 16B8 can detect antigens from diverse RABV strains. Baby hamster kidney (BHK)-21 cells (ATCC CCL10) infected with two reference RABV strains, challenge virus standard (CVS)-11 (ATCC VR959) and Evelyn-Rokitnicki-Abelseth (ERA), and the Korean isolate KGH were used to produce antigen slides of RABV. BHK-21 cells were grown in Dulbecco’s modified Eagle’s medium (Life Technologies, Waltham, MA, USA) supplemented with 10% fetal bovine serum (Life Technologies) inactivated at 56°C for 30 min, penicillin-streptomycin (Life Technologies), and l-glutamine (Life Technologies).

To evaluate any effect of the cell line type used for the test, antigen slides were produced with N2a cells according to the method described above. The cells were grown in Dulbecco’s modified Eagle’s medium-high glucose (Life Technologies) supplemented with 10% inactivated fetal bovine serum and penicillin-streptomycin.

Rabies antigen slides were prepared to contain 3,000 cells/well using each cell type infected with each virus strain for 48 h. Rabies direct fluorescent antibody (DFA) reagent (Millipore, Billerica, MA, USA), which is commercialized as a diagnostic antibody and commonly used for the detection of RABV antigen, was used as a positive control.

For the IFA, KGH P 16B8 was diluted with phosphate-buffered saline (PBS) including 1% bovine serum albumin at 1:100, and was added to each well of the antigen slides. The slides were incubated in a humid chamber at 37°C for 30 min and were washed by soaking in PBS for 10 min. After air drying, fluorescein isothiocyanate (FITC)-labeled anti-mouse immunoglobulin G conjugate (Jackson ImmunoResearch, West Grove, PA, USA) was diluted with PBS including 0.00025% Evans blue at 1:300 and was added to each well of the slides. The slides were incubated in a humid chamber at 37°C for 30 min and washed by soaking in PBS for 10 min. After mounting with a cover glass, the slides were observed within 2 h under a fluorescence microscope Axioskope2 (Zeiss, Oberkochen, Germany) at a magnification of 400×.

To evaluate the RABV detection capacity of 16B8-Alexa, its detection limit was measured using a RABV antigen slide with DFA reagent as the positive control. 16B8-Alexa was serially diluted five times from 1:100 to 1:12,500 with PBS including 0.00025% Evans blue. It was then placed on each well of the slides that were incubated in a humid chamber at 37°C for 30 min and washed by soaking in PBS for 10 min. After mounting with a cover glass, the slides were observed within 2 h under a fluorescence microscope at a magnification of 400×.

### Evaluation of the utility of the mAb in the RFFIT

#### Serum samples

A total of 414 human serum samples were used for this evaluation: 392 specimens were those deemed suitable for the experiment among clinical specimens that had been sent to the Division of Zoonoses of the KCDC between January 2006 and August 2015 to measure the neutralizing antibody titer using the RFFIT, and 22 serum samples were obtained from the Gyeongsang National University Hospital through the Korea Biobank Network (KBN) (S1 Table). The specimens requested to the Division of Zoonoses of the KCDC were obtained either from individuals whose immune responses were evaluated after PrEP or PEP, or from individuals whose neutralizing antibody titer indicated a requirement for continuous monitoring. Serum samples were also collected from individuals exposed to potentially rabid animals and patients with clinically suspected rabies. The specimens from the KBN were collected from patients aged between 0 and 76 years presenting symptoms of unspecified viral encephalitis, encephalitis, myelitis, and/or encephalomyelitis that are similar to those of rabies.

#### RFFIT

To evaluate the utility of 16B8-Alexa as a diagnostic antibody, RFFIT results obtained using DFA reagent were compared to those obtained using 16B8-Alexa according to the standard protocol suggested by the WHO [10]. The reference material anti-rabies human immunoglobulin (RAI; National Institute for Biological Standards and Control, UK) diluted to 2 IU/mL was used as the reference serum standard. For each experiment, the RAI solution was diluted to 0.5 IU/mL as the positive control and to several concentrations <0.1 IU/mL to serve as negative controls alongside the reference. All of the serum specimens were stored at −20°C until use, and were inactivated at 56°C for 30 min prior to the experiment.

The CVS-11 strain, which was adjusted to a focus-forming dose (FFD)_50_of 100 through titration, was used as the test virus in the RFFIT. N2a cells that had been incubated for 3–5 days were used at a passage number of less than 20 when counted from the passage upon obtainment; the cell density was adjusted to 1 × 10^5^cells/well.

Inactivated serum was serially diluted five-fold so that the final dilutions ranged from 1:5 to 1:625 and was placed in the wells of an 8-well chamber slide. A virus suspension equivalent to 100 FFD_50_ was added to each well and the slide was incubated at 35°C with 5% CO_2_ for 90 min for neutralization. After the incubation, N2a cells were added to each well and the slide was incubated at 35°C with 5% CO_2_ for 20 h. Subsequently, any growth medium remaining on the slide was removed, and the slide was washed once with PBS and fixed with 80% cold acetone.

For staining, the working solution of 16B8-Alexa diluted with PBS containing 0.00025% Evans blue at 1:200 was placed in each well of the slide. The slides were incubated in a humid chamber at 37°C for 30 min and then washed by soaking in PBS for 10 min. After mounting with a cover glass, the slides were observed within 2 h under a fluorescence microscope at a magnification of 200×. The experiment was performed in duplicate and the results were read twice by two different researchers; the average result was used for analysis.

The Reed and Muench formula was used for calculation of the virus-neutralizing antibody titer [11]. The results are expressed in IU/mL rounded to two decimal places.

### Statistical analysis

The results obtained through the RFFIT using the total of 414 clinical specimens were analyzed according to the value of 0.5 IU/mL, which was established as an indication of adequate vaccination in humans at risk of rabies exposure [1]. Accordingly, for qualitative analysis, the RFFIT results were classified into two categories: a value of 0.5 IU/mL or higher was considered positive, and 0–0.49 IU/mL was considered negative. The applicability of 16B8-Alexa to the RFFIT was evaluated in terms of its diagnostic sensitivity, specificity, positive predictive value (PPV), and negative predictive value (NPV). The correlation coefficient between the results of the RFFIT using 16B8-Alexa and the DFA reagent was evaluated by Pearson's correlation analysis. A P-value <0.01 was regarded to indicate statistical significance. IBM SPSS Statistics, Version 21.0 was used for all statistical analyses.

## Results

### Evaluation of the KGH P-specific mAb

#### Optimization of conditions for target antigen expression

**Fig 1:**
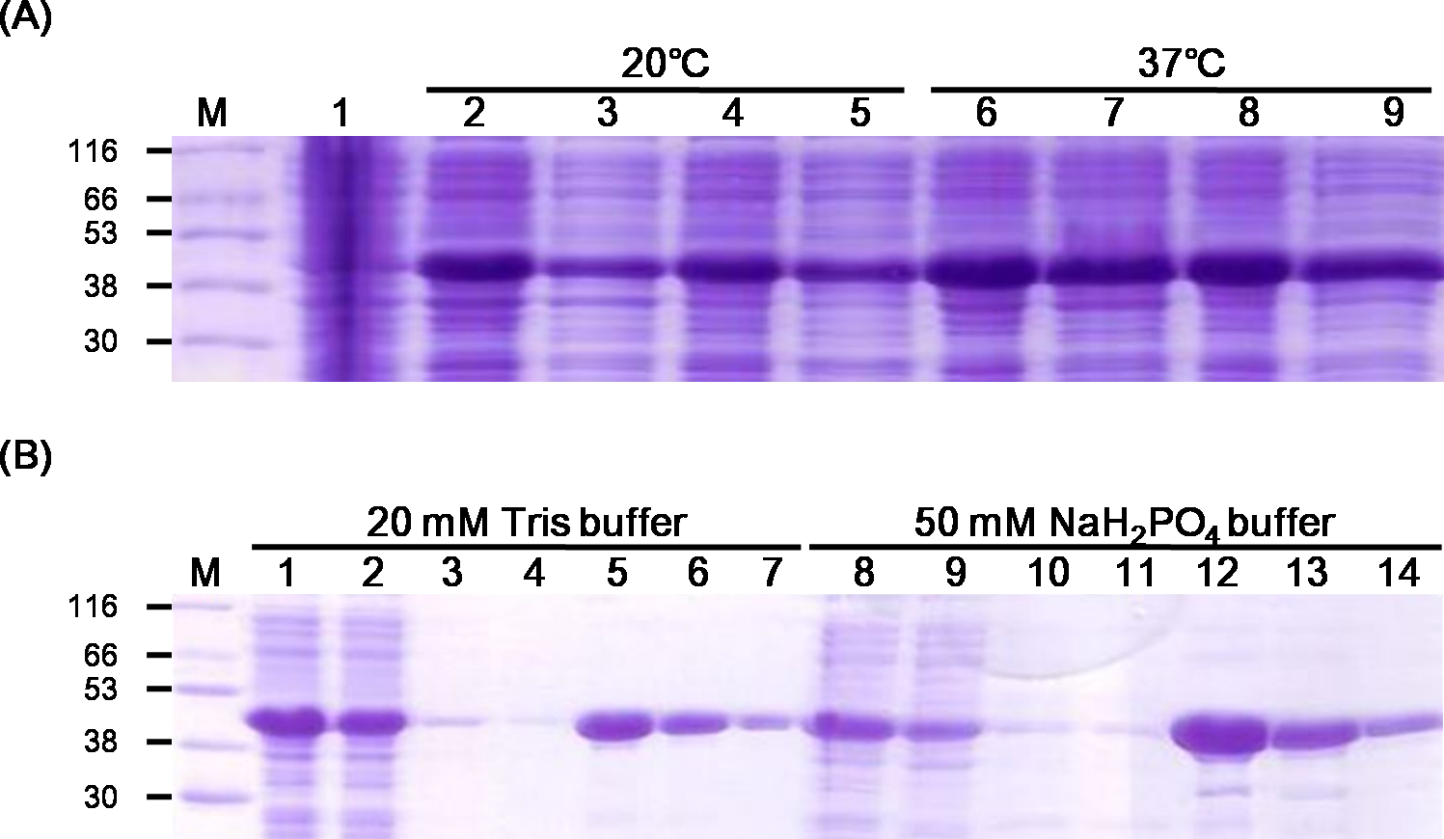
Optimization of KGH P protein expression in BL21(DE3) *E*. *coli* and purification. (A) Protein expression according to temperature. M, protein size marker; 1, uninduced total cell lysate; 2 and 6, total cell lysate prepared from cells induced with 0.4 mM IPTG using 20 mM Tris lysis buffer; 3 and 7, total cell lysate prepared from cells induced with 0.4 mM IPTG using 50 mM NaH_2_PO_4_lysis buffer; 4 and 8, cell lysate supernatant prepared from cells induced with 0.4 mM IPTG using 20 mM Tris lysis buffer; 5 and 9, cell lysate supernatant prepared from cells induced with 0.4 mM IPTG using 50 mM NaH_2_PO_4_lysis buffer. (B) Protein expression according to buffer composition. M, protein size marker; 1 and 8, cell lysate supernatant; 2 and 9, flow-through; 3, 4, 10, 11, wash; 5–7, 12–14, elution.

Soluble protein was expressed in all cell types under all conditions tested except when cells were transfected with BL21(DE3)pLysS at an IPTG concentration of 0.4 mM (S1 Fig). The KGH P protein expression levels according to the temperature and buffer composition are presented in Fig 1. The target protein was expressed at both temperatures tested (Fig 1A), and 20 mM Tris was found to be the best buffer for purifying the expressed protein by Ni-NTA His-tag affinity chromatography (Fig 1B). Protein expression was verified by SDS-PAGE. Based on the results, the optimal conditions for target antigen expression in BL21(DE3) *E*. *coli* competent cells were determined to be induction with 0.4 mM IPTG at 20°C overnight with shaking at 110rpm.

#### Reactivity of KGH P 16B8

**Fig 2:**
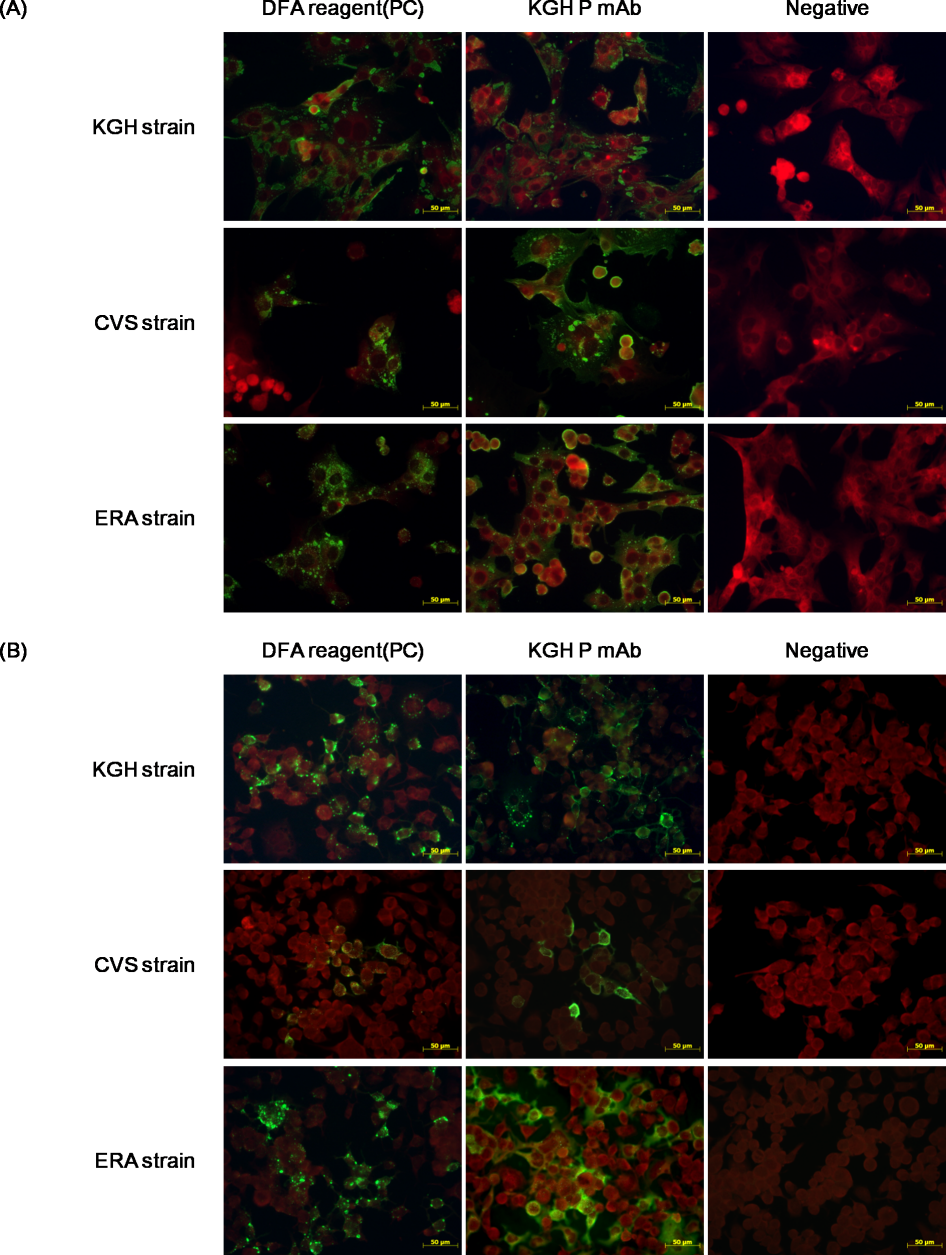
IFA reactivity according to RABV strain and infected cell type. (A) BHK-21 cells and (B) N2a cells. The cells were infected with the KGH, CVS-11, and ERA strains for 48 h. The results were observed under a fluorescence microscope at a magnification 400×.

IFA reactivities of KGH P 16B8 with various strains of RABV amplified in BHK-21 cells and N2a cells are presented in Fig 2A and 2B, respectively. The results demonstrated that the mAb could detect viruses of the KGH strain, which was used to produce the mAb, as well as of the reference strains CVS-11 and ERA. Virus was detected in both N2a and BHK-21 cells, indicating no effect of cell type.

#### Reactivity of 16B8-Alexa

**Fig 3:**
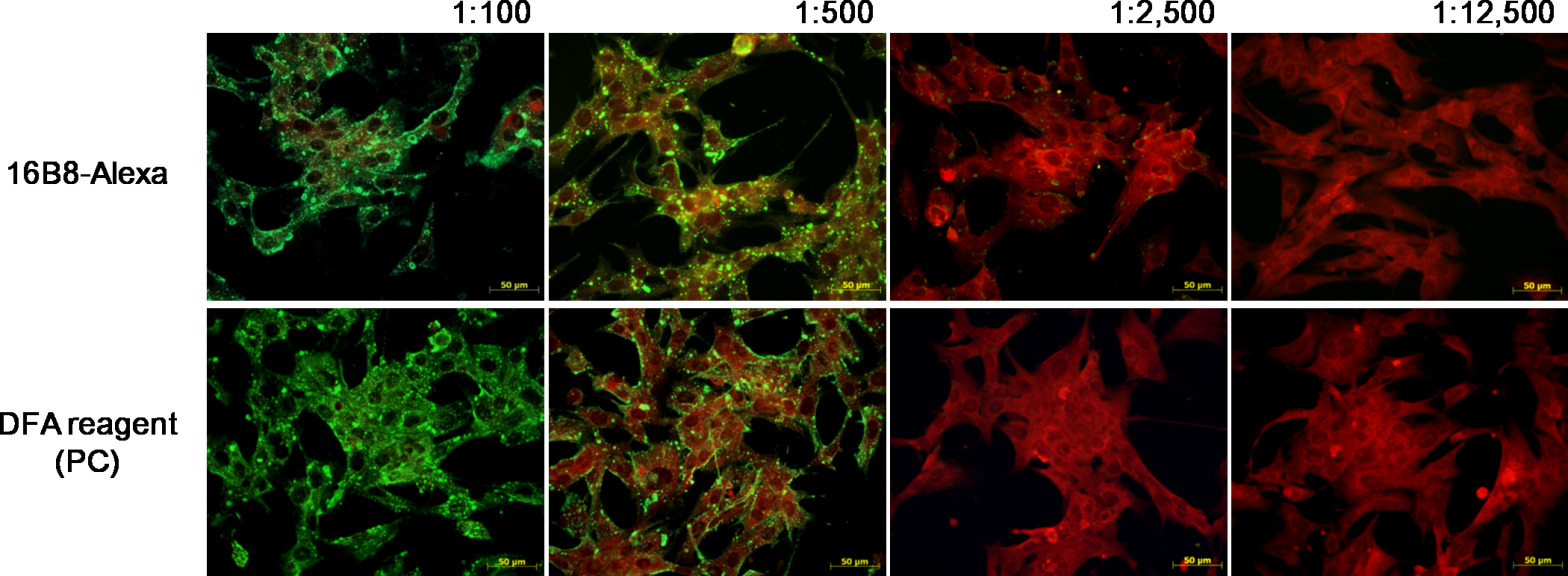
Comparison of the detection limits of the 16B8-Alexa diagnostic antibody with that of the DFA. (A) 16B8-Alexa and (B) commercial DFA reagent were five-fold serially diluted in PBS containing 0.00025% Evans blue to dilutions ranging from 1:100 to 1:12,500. Fluorescence microscopic images are shown at a magnification of 400×.

The limit of detection for RABV with 16B8-Alexa was determined in comparison to the reactivity of the DFA reagent. Virus detection with 16B8-Alexa was possible until a dilution ratio of 1:2500, and was not inferior to the detection ability of the DFA reagent used as a positive control; the results indicated that the IFA using 16B8-Alexa displayed a limit of detection when the serum was diluted to 1:500 (Fig 3).

When virus infected cells were stained with DFA reagent, a specific granule morphology with an apple-green color was observed. By contrast, a clear specific granule morphology was observed when the infected cells were stained with 16B8-Alexa.

### Effectiveness of 16B8-Alexa in the RFFIT

The RFFIT was conducted using 414 clinical serum specimens to evaluate the utility of 16B8-Alexa as a diagnostic mAb (S1 Table). The results of the RFFIT using 16B8-Alexa were compared to those obtained using the DFA reagent. Reference serum was used in each experiment to calculate the antibody titers (IU/mL). High sensitivity and specificity values of 98.67% and 99.47%, respectively, were obtained in the RFFIT using 16B8-Alexa. In addition, there was very good (99.03%) agreement between the results obtained with 16B8-Alexa and DFA; the PPV and NPV of RFFIT using 16B8-Alexa was 99.55% and 98.42%, respectively (Table 1). Discordant results were observed in four samples for which the titer ranged between 0.42 and 0.53 IU/mL.

**Table 1:** RFFIT results using DFA reagent and 16B8-Alexa.

		DFA reagent results		
		Positive	Negative	Total
**16B8-Alexa** **results**	**Positive**	223	1	224
	**Negative**	3	187	190
	**Total**	226	188	414

**Fig 4:**
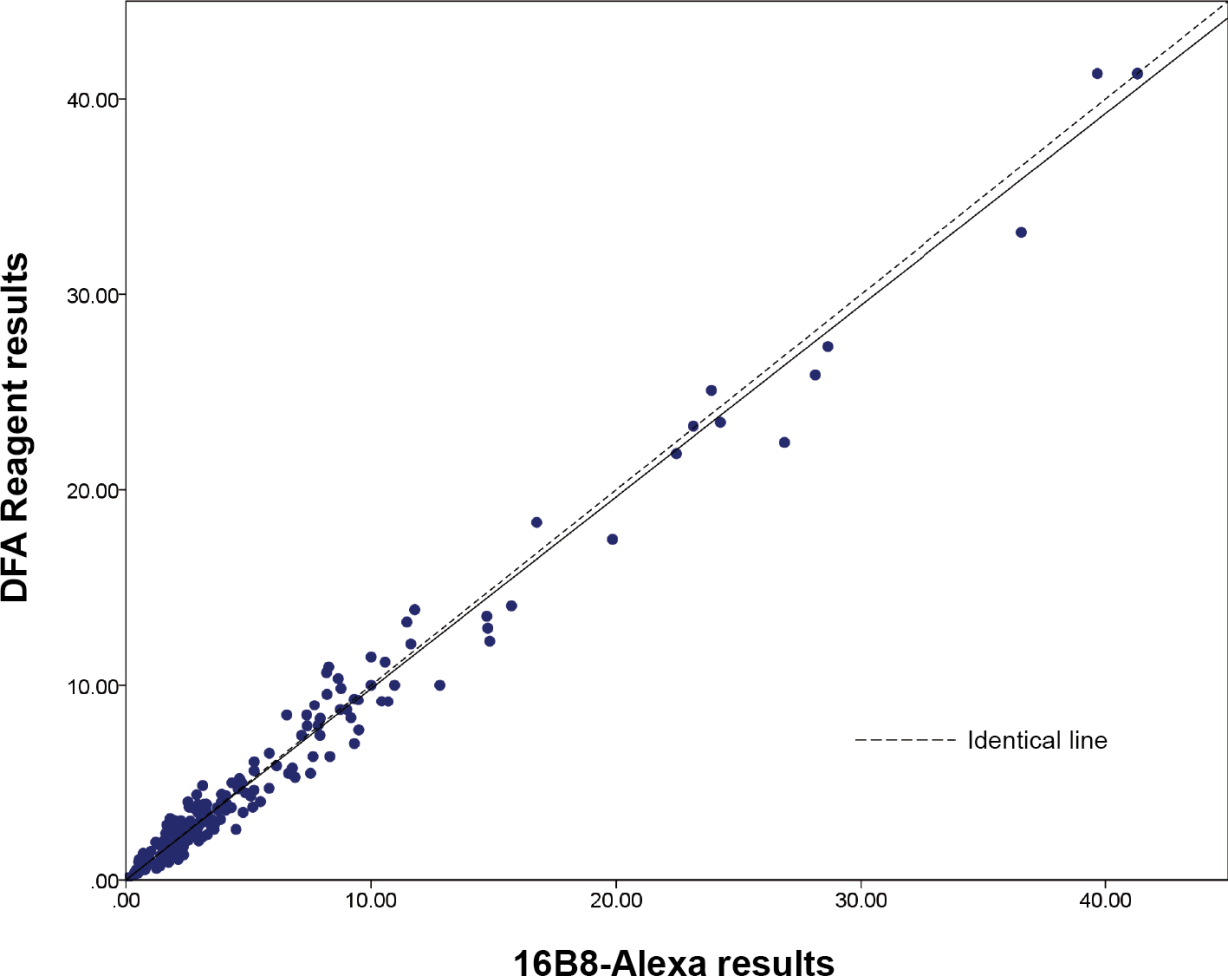
Correlation of RFFIT results of 414 clinical specimens, using DFA reagent and 16B8-Alexa. Significant correlation was observed (r = 0.995, *p*<0.001).

Owing to the nature of the experiments, the detection limit varied between experiments. In this study, the measurable minimum titer was 0.02 IU/mL. Therefore, as it was not possible to measure the IU value in cases without a neutralizing antibody titer, immeasurably low values were substituted with 0.01 IU/mL in correlation analysis to minimize any impact of this uncertainty. For the same reason, the measurable maximum in this study was set to 41.29 IU/mL, and data for samples with immeasurably high antibody titers were substituted with 41.30 IU/mL in correlation analysis to minimize any impact of this uncertainty. Pearson's correlation analysis showed a significantly high level of correlation (r = 0.995, *p*<0.001) between the DFA reagent assay results and the 16B8-Alexa assay results (Fig 4).

## Discussion

In this study, we developed a new mAb that reacts specifically with the RABV P protein and confirmed its applicability to the RFFIT. Numerous previous studies have reported antibodies developed as diagnostic reagents for rabies detection that target the N protein [12,13] or G protein [14,15]. N protein is related to the immune response and pathogenicity in the host. Because of its high inter-species conservation and its high-level production upon infection, N protein is frequently used as a target protein in rabies diagnosis. G protein is a representative membrane protein, and is the only antigen that induces virus-neutralizing antibodies. The amount of each type of protein in the virus varies slightly among studies. Nonetheless, N protein is generally reported to be present in the largest amount (1325 or 1800 copies), followed by P protein (691 or 950 copies) [16]. P protein is a multi-functional protein interacting with N (N-P) and is a key component and regulatory protein of the P-L complex for viral genome replication [17]. P protein is detected in both the virus and virus-infected host cells, in two forms: a major hypo-phosphorylated 37-kDa form and a minor hyper-phosphorylated 40-kDa form [18]. mAbs targeting P protein have been developed in some studies [19,20]. In this study, 16B8-Alexa was developed using a KGH P protein-specific mAb. 16B8-Alexa detected the viral antigen in two types of cells infected with RABV, with no significant difference in detection ability as compared to existing diagnostic reagents. Specific granule morphology was also clearly observed in infected cells. These findings confirmed that 16B8-Alexa can be applied as a diagnostic reagent to specifically target the RABV P protein.

In the preliminary experiments, the sensitivity of Alexa Fluor was found to be higher when used as a marker for labeling KGH P 16B8 than that of FITC. Although fluorescein is more frequently used for mAb development, Alexa Fluor exhibits stronger fluorescence and is less pH-sensitive; thus, it is a safety-certified fluorescent particle [21], and was chosen as the fluorescent marker in this study. Moreover, the detection limit of 16B8 Alexa in the RFFIT was not inferior to that of existing diagnostic reagents.

To date, 14 virus species, including RABV, have been identified in the genus *Lyssavirus* [22]. Based on phylogenetic analysis, these viruses are categorized into seven major genotypes, with the prototype RABV belonging to genotype 1 [23]. In general, the prototype RABV belonging to genotype 1 is the main causal agent of rabies, but the disease can also rarely be caused by other lyssaviruses [24]. For this reason, most of the commercial antibodies available for rabies diagnosis are designed to detect rabies-related lyssaviruses along with RABV most of the cases [25]. The majority of diagnostic laboratories utilize commercial reagents [26], whereas some utilize in-house products [15, 27, 28]. Both polyclonal antibodies and mAbs, which are specific to the entire virus or to the nucleocapsid protein and are conjugated with a fluorophore, can be used for rabies diagnosis. The Office International des Epizooties suggests that the sensitivity and specificity of any produced fluorescent antibody used in the fluorescent antibody test (FAT) should be verified through thorough validation and should be able to detect other lyssaviruses as well [29].

In this study, the ability of 16B8-Alexa to detect the prototype RABV was confirmed; however, it was not possible to confirm its ability to detect rabies-related lyssaviruses owing to experimental limitations. This remains a constraint in applying 16B8-Alexa to the testing of all lyssaviruses that could give rise to rabies symptoms. The main testing method of focus in our study was the RFFIT, and 16B8-Alexa was confirmed to detect the CVS-11 strain, which is the only RABV strain recommended for use in the RFFIT. Therefore, 16B8-Alexa can be used in the RFFIT to measure the neutralizing antibody titer in the serum of vaccinated individuals.

The applied significance of this work has been demonstrated, as the utility of 16B8-Alexa was evaluated using 414 clinical specimens. In reference to 0.5 IU/mL as the marker concentration for a sufficient vaccination effect suggested by the WHO [30], 16B8-Alexa demonstrated high sensitivity and specificity, and the agreement between the two assay methods evaluated using 16B8-Alexa or DFA reagent was also significantly high. Furthermore, the PPV and NPV were both very high. Therefore, we suggest that 16B8-Alexa can be utilized in the RFFIT for the measurement and diagnosis of rabies neutralizing antibody titers.

Discordant results using 16B8-Alexa and DFA were observed in four samples, all of which had titers close to the limit of 0.5 IU/mL and were thus considered to represent variations within the acceptable range. These discordant results could be associated with issues of experimental reproducibility rather than an inherent difference in the efficiency of the diagnostic reagent. The RFFIT applies relatively loose restrictions for standardization, as it is a cell-based bioassay using biological materials such as live viruses [31].

The tested clinical specimens included 22 serum samples of patients obtained from the KBN; seven of the patients had viral encephalitis and 15 had encephalitis, myelitis, and encephalomyelitis. All of these patients presented symptoms similar to those of rabies, including fever (68.18%), vomiting (45.45%), convulsion/numbness (40.90%), and headache (36.36%). Therefore, these samples were used to test the cross-reactivity of 16B8-Alexa. In the majority of patients with rabies, central nervous system infection symptoms such as encephalitis or encephalomyelitis are observed. Therefore, differential diagnosis is required using the neutralizing antibody titer of the sera of patients presenting such symptoms [32]. In the present study, the specificity using these specimens was 100%.

The 16B8-Alexa developed in this study was shown to react strongly with both fixed and street RABV strains, and as it targets the P protein of RABV, the reagent is expected to be useful in Korea with high potential risk of bat-related lyssaviruses. Furthermore, as a reagent developed and available in the destination area, the following problems can be overcome: elimination of the economic burden associated with import; reduction in the time required for completing certain procedures by up to 25% of the previous level; and avoidance of any unnecessary efforts for passing through the complicated import procedures.

As potential advantages, 16B8-Alexa can be applied to a diverse array of other testing methods for prototype RABV detection beyond those involving fluorescence microscopy, simply by replacing the marker substance with another. As 16B8-Alexa is a RABV P protein-specific mAb, its utility will also be valuable for conducting basic immunological research to better understand the functions of P protein. With future studies to evaluate its detection ability for other lyssaviruses and investigations into its robustness and stability, it is expected that 16B8-Alexa can resolve the current issues of the limited accessibility of existing diagnostic reagents, and will serve as a valuable alternative diagnostic reagent free of economic constraints.

## Supporting information

S1 Table Samples used for the evaluation of the utility of the 16B8-Alexa and the results of RFFIT. (XLSX)

S1 Fig Optimization results for KGH P protein expression according to type of competent cell and temperature. (PDF)
