# Supplementary material for: Development and evaluation of an anti-rabies virus phosphoprotein-specific monoclonal antibody for detection of rabies neutralizing antibodies using RFFIT
Source: PLoS Negl Trop Dis. 2017 Dec 21;11(12):e0006084. doi: 10.1371/journal.pntd.0006084 (PMC5755941; doi:10.1371/journal.pntd.0006084)
Supplement: S1 Fig — (PDF) [file pntd.0006084.s002.pdf]

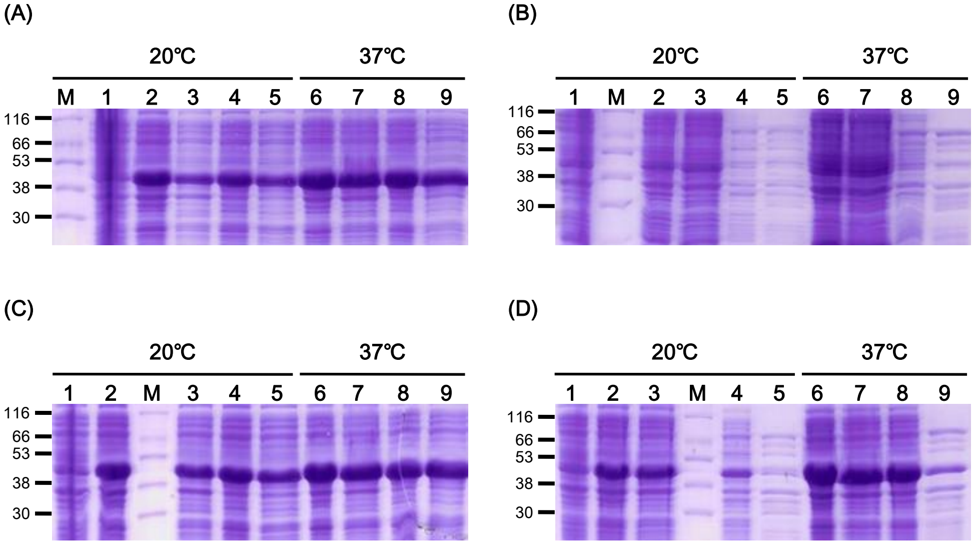

S1 Fig. Optimization results for KGH P protein expression according to type of competent cell and temperature. Total lysates were separated by SDS-PAGE and visualized by coomassie staining. (A) BL21(DE3), (B) BL21(DE3)pLysS, (C) BL21 codon-plus RIL and (D) Rosetta(DE3). M, protein size marker; 1, uninduced total cell lysate; 2 and 6, total cell lysate induced with 20 mM Tris lysis buffer; 3 and 7, total cell lysate induced with 50 mM NaH_2PO_4lysis buffer; 4 and 8,cell lysate supernatant induced with 20 mM Tris lysis buffer; 5 and 9, cell lysate supernatant induced with 50 mM NaH_2PO_4 lysis buffer.
